# Supplementary material for: Quantitative Monitoring of Mycelial Growth of Aspergillus fumigatus in Liquid Culture by Optical Density
Source: Microbiol Spectr. 2022 Jan 5;10(1):e00063-21. doi: 10.1128/spectrum.00063-21 (PMC8729762; doi:10.1128/spectrum.00063-21)
Supplement: SUPPLEMENTAL FILE 1 — Supplemental material. Download SPECTRUM00063-21_Supp_1_seq7.pdf, PDF file, 1.2 MB [file spectrum00063-21_supp_1_seq7.pdf]

Supplemental Material

Quantitative monitoring of mycelial growth of *Aspergillus fumigatus* in liquid culture by optical density

Ken Miyazawa, Takashi Umeyama, Yasutaka Hoshino, Keietsu Abe, and Yoshitsugu Miyazaki*

*Correspondence: Y. Miyazaki, ym46@niid.go.jp

Table S1. Primers used in this study.

Primer name	Sequence (5′ to 3′)
Afags1-LU	AGAGTCGAGGAAATCACCGTTG
Afags1-LL+hph	GGCCATCTAGGCCAGAATATAGGGAAATTGCACAACCGAC
Afags1-RU+hph	GGCCTGAGTGGCCTCCGAAGCTAGCTACGTGAACCTG
Afags1-RL	CAGGATCGAGGTAATCAGGTATGTG
397-5	GAGGCCACTCAGGCCGATATCACC
397-3	CTGGCCTAGATGGCCGTCGACAAC
IF1-lox66-hph-Fw	TACCGTTCGTATAATGTATGCTATACGAAGTTATCTGGCCTA GATGGCCGTCGACAAC
IF2-PxynG2-Fw	GGCCTGAGTGGCCTCCCTCGTACTCGATGACGGCTTC
IF2-lox71-TagdA-Rv	TACCGTTCGTATAGCATACATTATACGAAGTTATGTTTCGAG GGGTACCTGGTG
IF3-lox71-pUC19	GCTATACGAACGGTATCTAGAGTCGACCTGCAGGCATG
IF3-lox66-pUC19	ATTATACGAACGGTACCGGGTACCGAGCTCGAATTC
M13-47	CGCCAGGGTTTTCCCAGTCACGAC
RV-M	GAGCGGATAACAATTTCACACAGG
Afags1-LL-M13	GTCGTGACTGGGAAAACCCTGGCGAATATAGGGAAATTGC ACAACCGAC
Afags1-RU-M13	CCTGTGTGAAATTGTTATCCGCTCCGAAGCTAGCTACGTGA ACCTG
Afgtb3-LU	GGCATGGCATCGTACTTCGTG
Afgtb3-LL+hph	GGCCATCTAGGCCAGGACGTCCAAAGATCCGTACTGTG

Afgtb3-RU+hph	GGCCTGAGTGGCCTCCATCGCGCTGATGATCTCTCTG
Afgtb3-RL	CAAGGCAAGACTCCAGACCTG

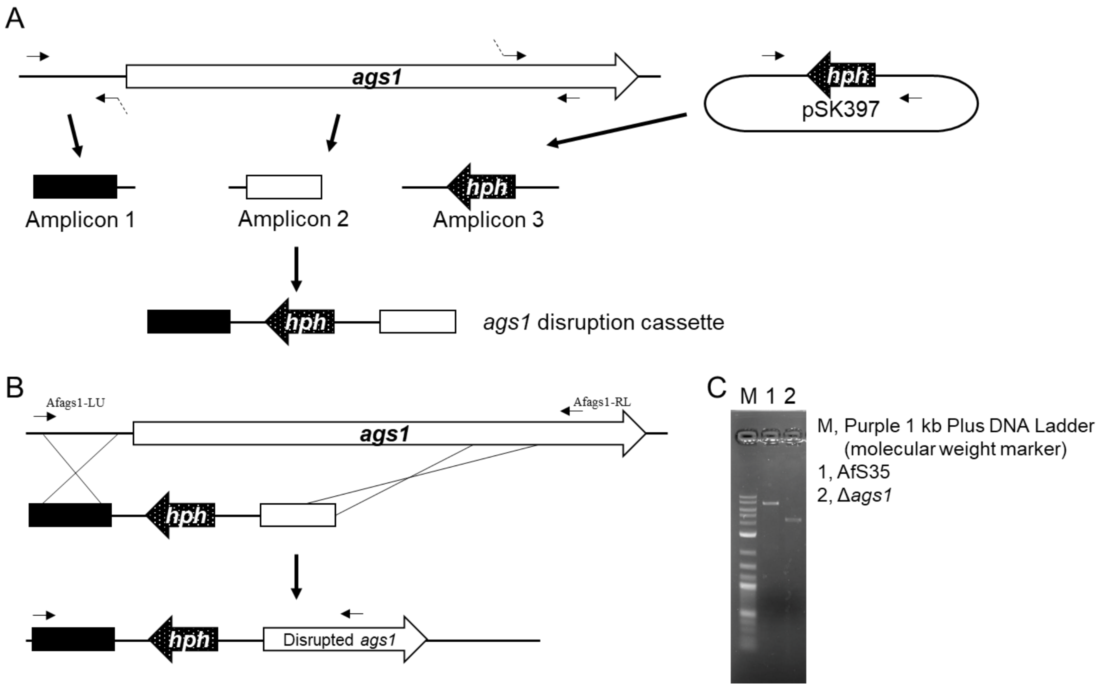

Figure S1. Construction of the Δ*ags1* strain. (**A**) Scheme of construction of the *ags1* disruption cassette. (**B**) Strategy for replacement of the disrupted *ags1* gene with the hygromycin resistance marker *hph*. (**C**) PCR analysis of *ags1* gene disruption with the primers indicated in (**B**).

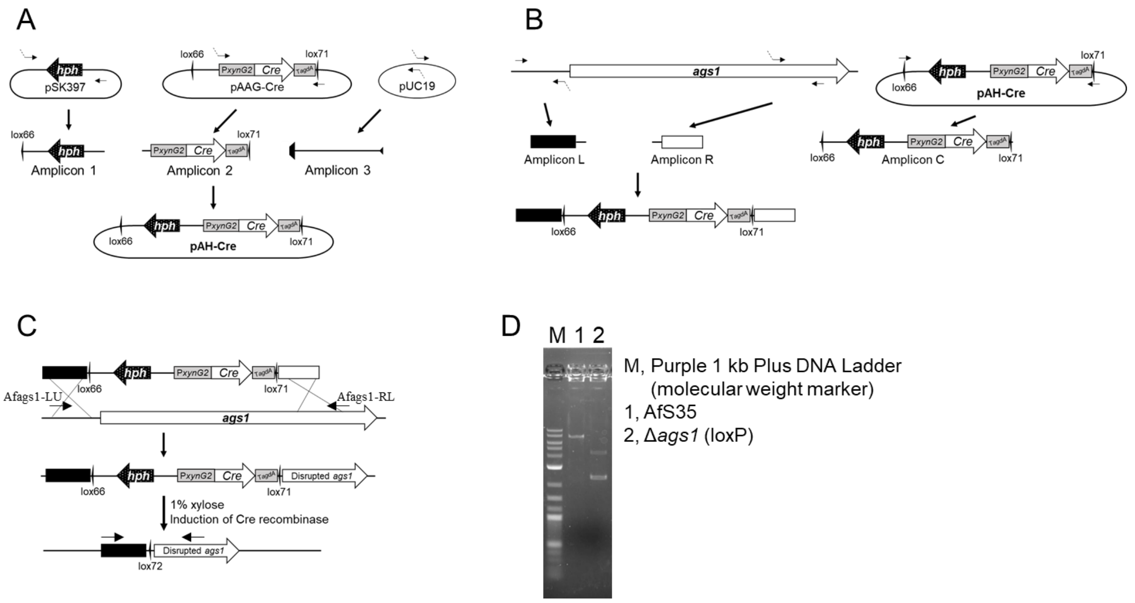

Figure S2. Construction of the Δ*ags1* (loxP) strain by using the Cre/mutant *loxP* marker recycling system. (**A**) Construction of the pAH-Cre plasmid. Fragments containing *hph*, P*xynG2* and *Cre*, and *ampR* and ori were amplified and fused using a NEBuilder HiFi DNA Assembly kit. (**B**) Construction of the *ags1* gene disruption cassette containing the Cre/mutant *loxP* marker recycling system. (**C**) Strategy for gene replacement to disrupt *ags1*. The PCR-amplified cassette was used to transform the AfS35 strain. Candidate strains were isolated on the basis of hygromycin resistance. Then the *Cre* gene was induced by culture on AMM medium containing 1% xylose, resulting in marker cassette excision. (**D**) PCR analysis of *ags1* gene disruption using the primers indicated in (**C**).

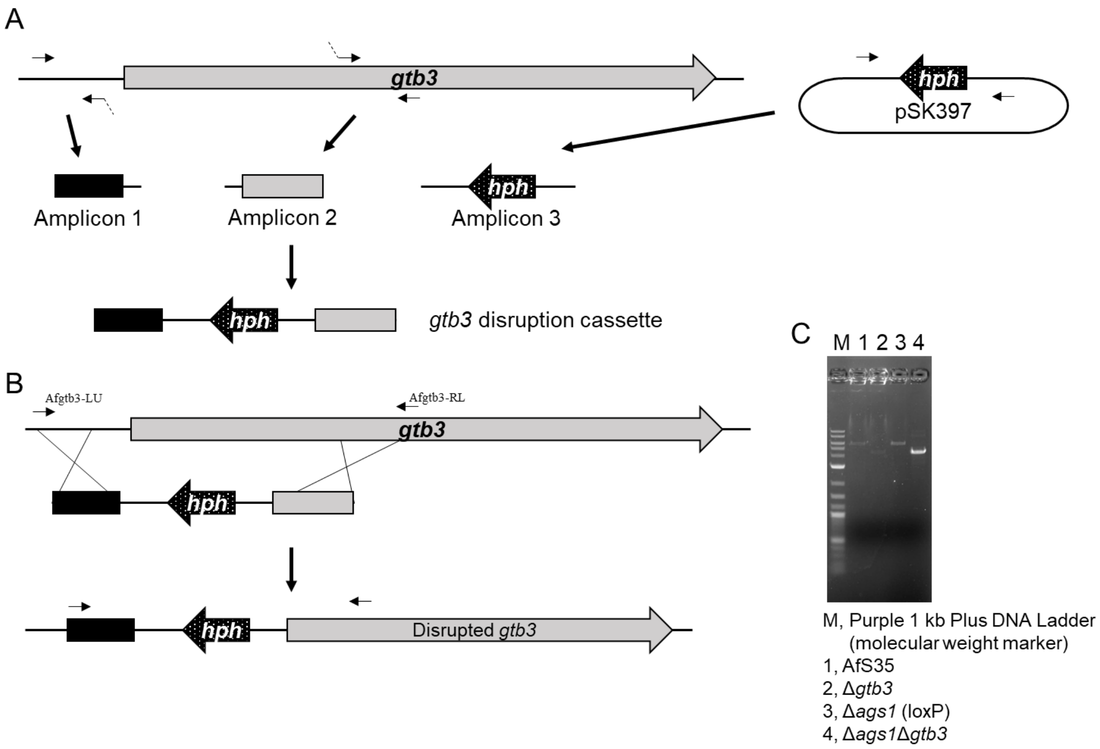

Figure S3. Disruption of the *gtb3* gene to generate single (Δ*gtb3*) and double (Δ*ags1*Δ*gtb3*) mutants. (**A**) Scheme of construction of the *gtb3* disruption cassette. (**B**) Strategy for replacement of the disrupted *gtb3* gene with the hygromycin resistance marker *hph*. (**C**) PCR analysis of *gtb3* gene disruption with the primers indicated in (**B**).

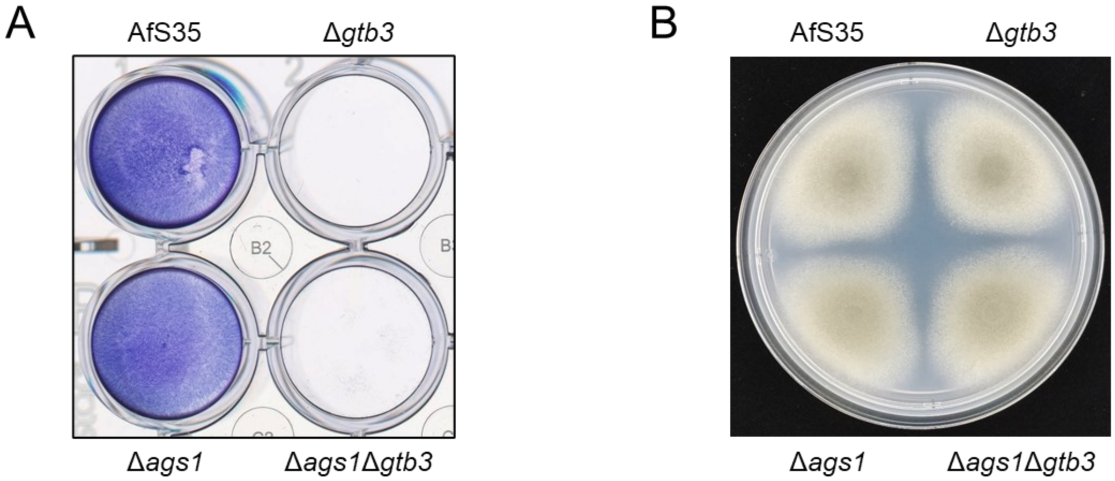

Figure S4. Biofilm formation and radial growth of the AfS35, Δ*ags1*, Δ*gtb3*, and Δ*ags1*Δ*gtb3* strains. (**A**) Biofilm formation on polystyrene plates. Conidia (1×10^4) of each strain were inoculated into AMM medium in a 24-well plate and incubated at 37°C for 24 h, after which the biofilms were washed and stained with crystal violet. (**B**) Conidia (1×10^4) of each strain were inoculated at the center of an AMM agar plate and incubated at 37°C for 4 days.

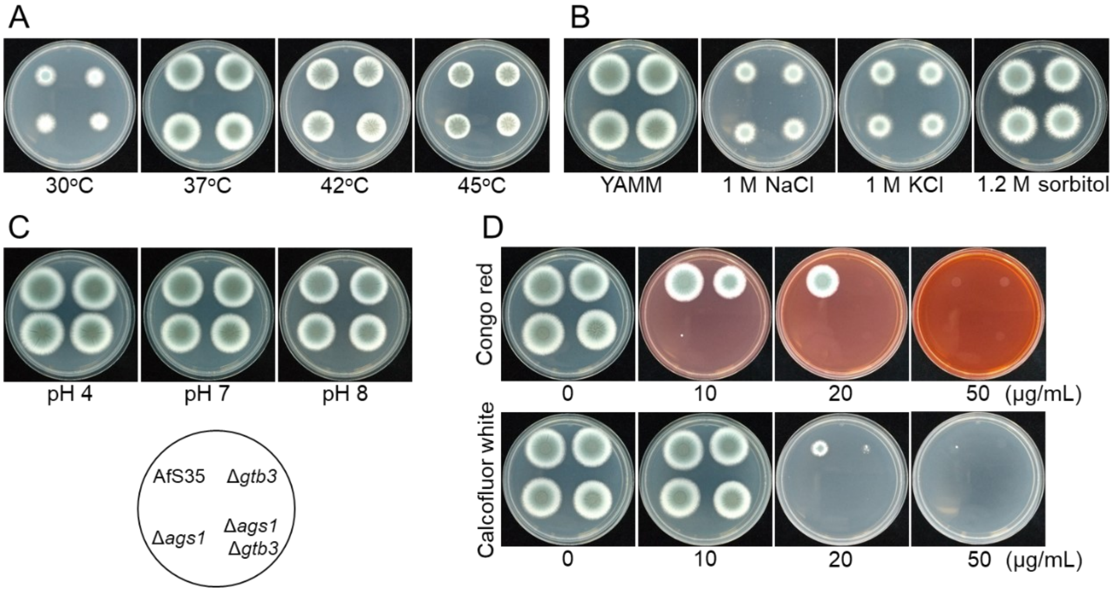

Figure S5. Growth of the AfS35, Δ*ags1*, Δ*gtb3*, and Δ*ags1*Δ*gtb3* strains on plates under stress conditions. Conidia ((**A**–**C**) 1.0×10^4; (**D**, **E**), 1.0×10^5) were inoculated onto YAMM plates as indicated at the bottom left. and grown for 48 h. Plates were incubated (**A**) at the indicated temperatures; (**B**) in the presence of 1 M NaCl, 1 M KCl, or 1.2 M sorbitol in the medium at 37°C; (**C**) at the indicated pH at 37°C; (**D**) in the presence of congo red at 37°C; or (**E**) in the presence of calcofluor white at 37°C.

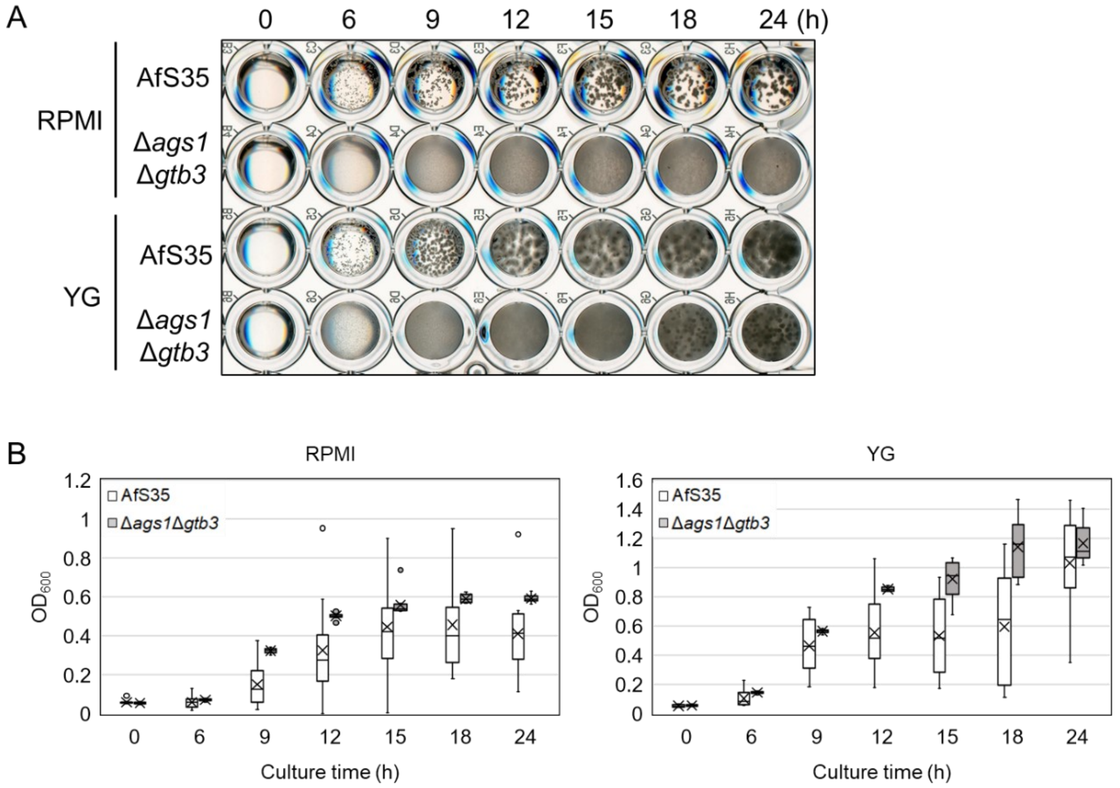

Figure S6. Turbidity of the Δ*ags1*Δ*gtb3* strains cultured in YG and RPMI medium. (**A**) Image of a culture plate with strains. (**B**) Time course of OD_{600}. The values of OD_{600} values were calculated from 12 measurements per time point and are shown as by box plots. Lines in boxes indicate medians, and crosses indicate averages. Circles indicate outliers.

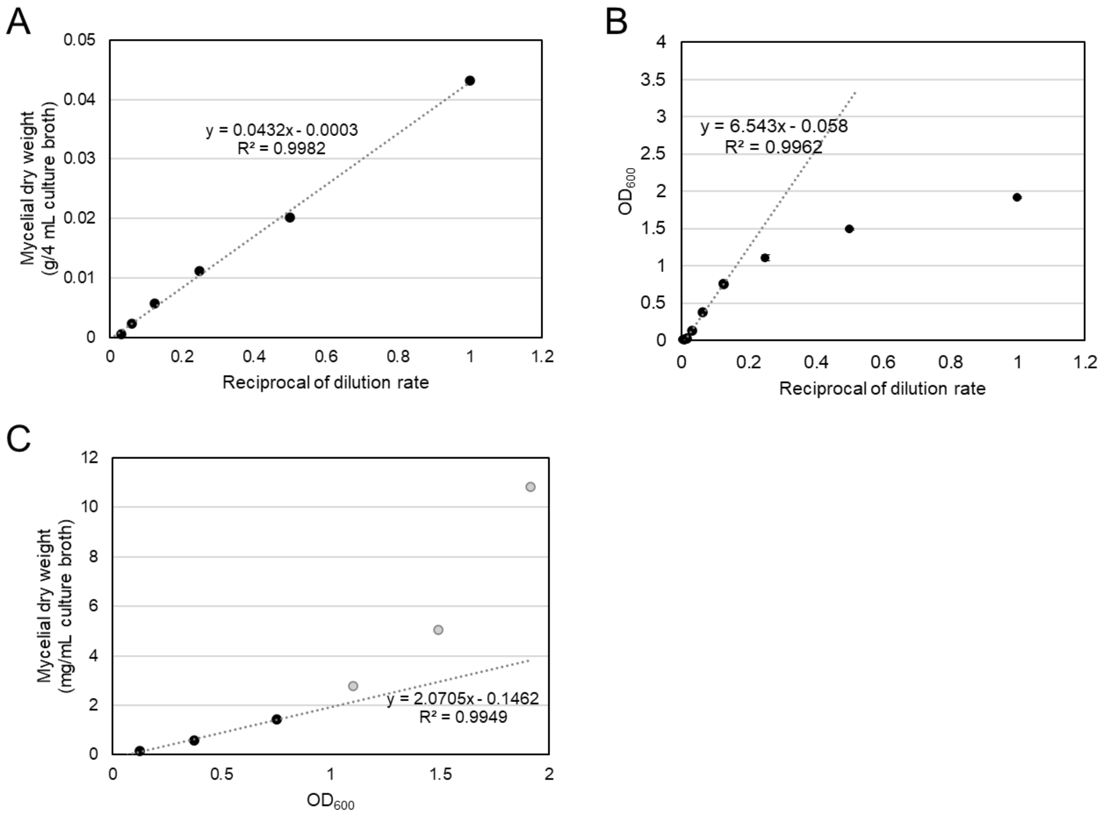

Figure S7. Linear regression analysis. Conidia (1.0×10^7/mL) of the Δ*ags1*Δ*gtb3* strain were inoculated into AMM liquid medium and rotated at 160 rpm at 37°C for 18 h. The grown mycelia were filtered, and a dilution series of mycelial suspension was prepared. (**A**) Reciprocal of dilution rate versus mycelial dry weight. (**B**) Reciprocal of dilution rate versus OD_{600}. (**C**) OD_{600} versus mycelial dry weight.

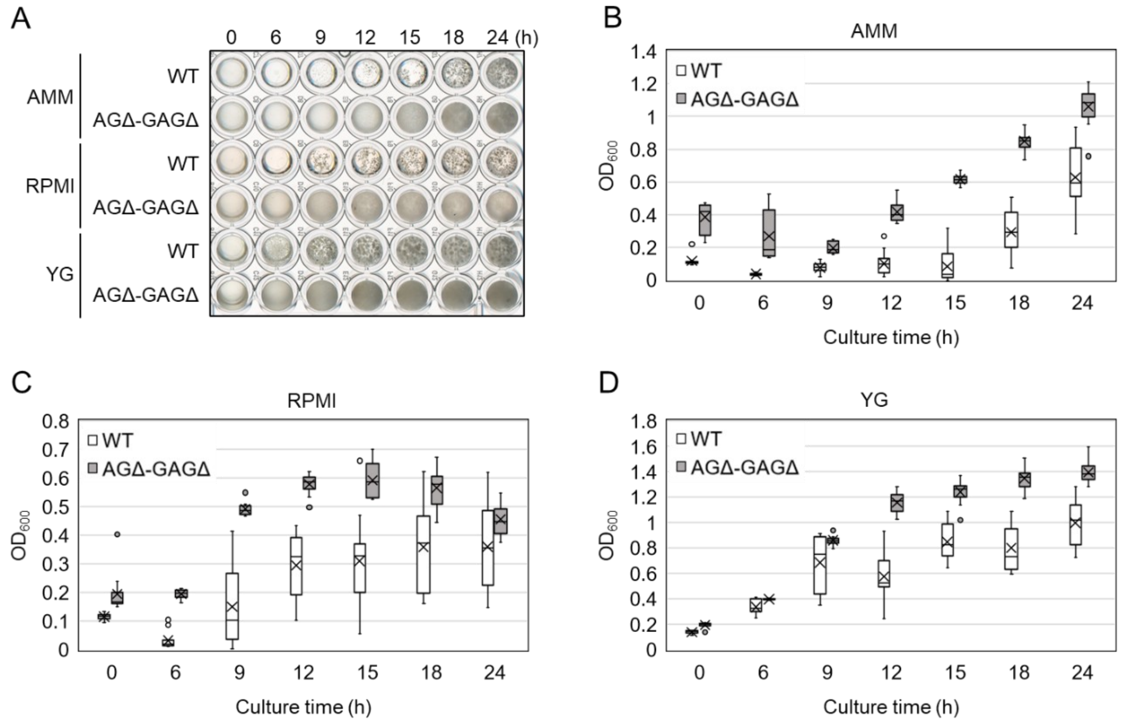

Figure S8. Optical density of the *Aspergillus oryzae* wild-type and AGΔ-GAGΔ strains. (**A**) Image of a culture plate with strains. Time course of OD_{600} in fungal cultures in (**B**) AMM, (**C**) RPMI, or (**D**) YG medium. The OD_{600} values were calculated from 12 measurements per time point and are shown as box plots. Lines in boxes indicate medians, and crosses indicate averages. Circles indicate outliers.

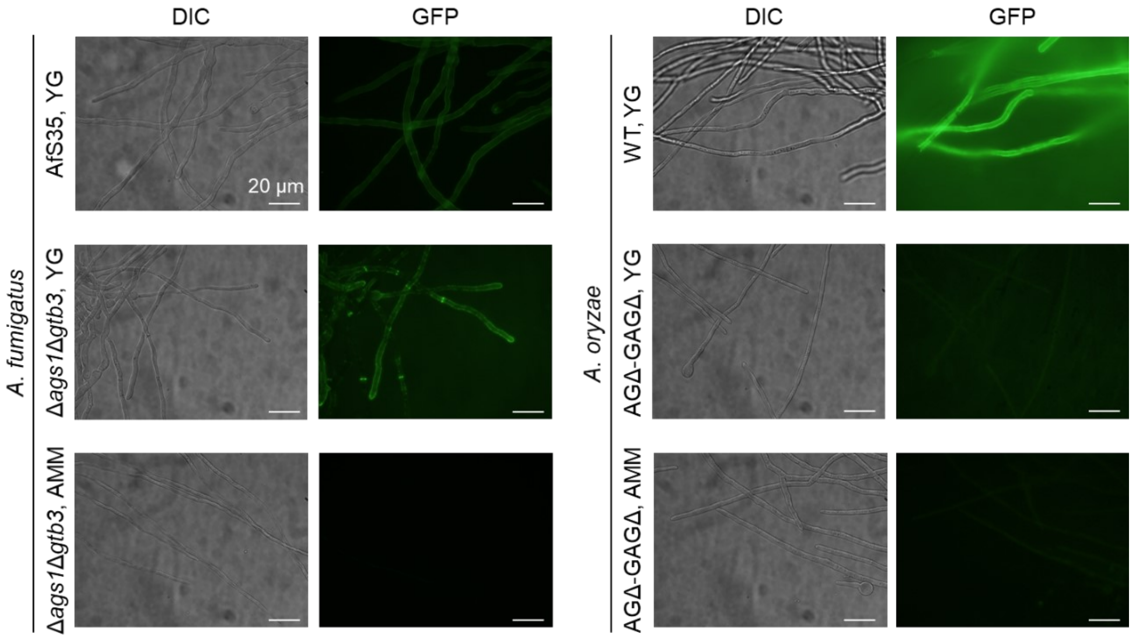

Figure S9. Labeling of hyphal cells with the α-1,3-glucan-binding domain of α-1,3-glucanase fused with GFP (AGBD–GFP). Hyphal cells were cultured in the indicated media for 24 h at 37°C (*A. fumigatus*) or 35°C (*A. oryzae*), fixed, and stained with AGBD–GFP to label α-1,3-glucan. DIC, differential interference contrast.
